# Can Agri-Food Waste Be a Sustainable Alternative in Aquaculture? A Bibliometric and Meta-Analytic Study on Growth Performance, Innate Immune System, and Antioxidant Defenses

**DOI:** 10.3390/foods11131861

**Published:** 2022-06-24

**Authors:** Filippo Bertocci, Giuseppe Mannino

**Affiliations:** 1 Department of Veterinary Medicine and Animal Productions, University of Naples Federico II, 80134 Naples, Italy; f.bertocci@studenti.unina.it; 2 Department of Life Sciences and Systems Biology, University of Turin, Via Quarello 15/a, 10135 Turin, Italy

**Keywords:** VOSviewer, forest plot, interleukin 1β (IL-1β), tumor necrosis factor α (TNF-α), growth performance, superoxide dismutase (SOD), catalase (CAT), antioxidant defenses, immune system

## Abstract

The agri-food industry generates a large amount of waste every year, which is both an environmental and economic problem, especially for the countries in charge of its disposal. Over the years, there has been a growing interest especially in plant waste, since they are rich in compounds with high nutritional and nutraceutical value. As a result, several scientific disciplines are investigating their alternative use in the formulation of dietary supplements for human or animal use, or as biostimulants for agricultural purposes. In this review, using a meta-analytical approach, we summarize the main and most recent findings related to the use of plant waste as potential ingredients in dietary supplementation for fish grown under controlled experimental conditions. In particular, in this review, it has been highlighted that plant waste may have not only positive effects on growth performance, but also beneficial effects on modulation of the innate immune system and antioxidant defenses. Finally, the bibliometric study and a mapping provide an overview of the recent publications, showing the research strength across the country, the number of potential collaborations among institutions, and the main research focus, demonstrating how this topic is growing in interest, especially in Europe.

## 1. Introduction

During the past century, the world population has increased three times more than the growth that occurred during the entire history of mankind [1,2]. In particular, it grew from 1.5 billion in 1900 to about 7.1 billion in 2003. According to current growth stages, it is expected that the world population will be about 9.4–10 billion in 2050 [3]. Since the population boost is linked to a greater demand for food, during the 20th century, it would not have been possible without a reliable rise in food production [4,5,6].

Land and water are two of the most important factors affecting food production, and they are in turn influenced not only by environmental changes but also by population growth [5,6,7,8,9]. For example, it has been estimated that from 2005 the lands devoted to agriculture or farming needs have been reduced by about 38%, resulting in an endowment of 0.76 ha per capita [6,7]. For this reason, modern strategies to support the large demand for food have focused mainly on finding innovative solutions to intensify the production process, rather than reclaim new areas. As a result, although areas committed to agriculture or livestock farming have decreased over time, total food production has sharply increased in the past 20 years, thanks in part to economic developments and technological advances [6,10]. On the other hand, the increase in food production is almost always followed by an increase in food waste. In particular, a recent study has shown that every year the processing of food for human consumption can generate up to 24% of waste during the different stages of the production chain, starting from cultivation, breeding, processing, storage, sale, up to the same domestic consumption [11,12]. While in under developing countries, food waste is mainly concentrated during the distribution phases, in developed countries, such as in Europe, food waste mainly occurs during the earlier steps, especially during the processing one [11,12]. A high level of food waste has negative consequences not only on the environment, but also on the society and economy of the countries charged with disposing of it according to current regulations [13]. Considering that the fruit and vegetable processing industry is one of the largest waste producers after domestic wastewater, finding new strategies for its reuse is one of the main goals of the modern circular economy. Recently, agricultural waste has been successfully used for the formulation of various products not only for agronomic purposes [3,14,15], but also for human or animal consumption [16,17,18,19]. Interest in this type of waste stems from the presence of bioactive compounds that can demonstrate great variability in biological actions. In particular, in the plant field, it has been shown that phytochemical compounds can increase the resilience of plants to different types of abiotic and biotic stresses, reducing the use of pesticides and fertilizers [8], while the same compounds can play important roles for the maintenance of redox balance, promotion of immune defenses, or prevention of some diseases in animals [19,20,21,22]. The use of plant waste as a potential ingredient in animal diets should not be underestimated. Indeed, because these molecules exert interesting properties, sometimes comparable to drugs, they can be a viable alternative to the administration of chemicals, hormones, or other exogenous substances that could compromise not only food quality but also the safety of food [19,20]. Moreover, analogous to human dietary supplements, it is reasonable to think that an animal diet supported by a constant and safe integration of plant-based bioactive molecules can be effective not only in preventing chronic diseases, but also in reducing stress factors, including those related to farming conditions [23].

## 2. Use of Agri-Food Waste in Aquaculture

In the past, it was always assumed that seas and oceans were an endless source of food. However, with the industrialization of fishing, wild fish stocks have become increasingly depleted [24]. Accordingly, to meet the demand of a growing global population, the aquaculture, particularly fish production, has rapidly intensified. For example, while aquaculture produced about 3 million tons of fish in the 1970s, today it is estimated that farmed fish exceeds 60 million tons. In particular, the Asian and Pacific regions dominate this scenario, accounting for 88.5% of the global production [25]. Moreover, global demand for aquaculture feed is expected to reach 70 million tons per year by 2025, doubling 2008 production and increasing almost 10 times that of 1995 [26,27].

The nutritional value of fish food is very high. Proteins derived from the consumption of fish products are more assimilable than meat ones, and the lack of connective tissues greatly facilitates digestive processes, making fish food more edible. Fish contains a considerable amount of calcium, which combined with its high vitamin D level and low cholesterol content makes it an extremely beneficial food for human health. Finally, fish is also a food rich in unsaturated fatty acids, especially ω3, essential fatty acids of limited distribution in both plant and animal kingdoms, which play an important role in the prevention of cardiovascular disease [28]. However, fish is a very expensive food. Its high cost depends on several factors, including (i) its easy perishability, which not only makes it difficult and very expansive to trade in inland regions, but also drastically reduces its shelf life after thawing; (ii) the obligation not to catch, or release after catching, fish that according to European directives are undersized; (iii) and the presence of local directives that prevent fishing to promote the restocking of the sea. The latter problems are obviously minimized by aquaculture techniques, to which however is added the additional cost of feeding fish. Currently, although the cost of farmed fish is much lower than that of wild fish, it has been calculated that more than 50% of the total cost of farmed fish is influenced by feed ingredients [29]. In this scenario, the use of food waste for fish farming would be a sustainable strategy not only to avoid the cost of organic waste disposal, but also a way to decrease the cost of feeding fish in aquaculture, and thus the final price to the consumer. However, can this practice also be a sustainable alternative in terms of food quality and safety? To answer this question, a bibliometric analysis has been conducted to understand the degree to which this topic has been investigated into the scientific literature. In addition, in order to evaluate the potential positive and/or negative effect derived from the use of agri-food waste as an ingredient for the fish diet, a meta-analysis study of data collected from articles published in the past 10 years that met the predetermined inclusion criteria has been performed.

## 3. Bibliometric Mapping of the Experimental Use of Agri-Food Waste for Fish Nutrition

**Figure 1:**
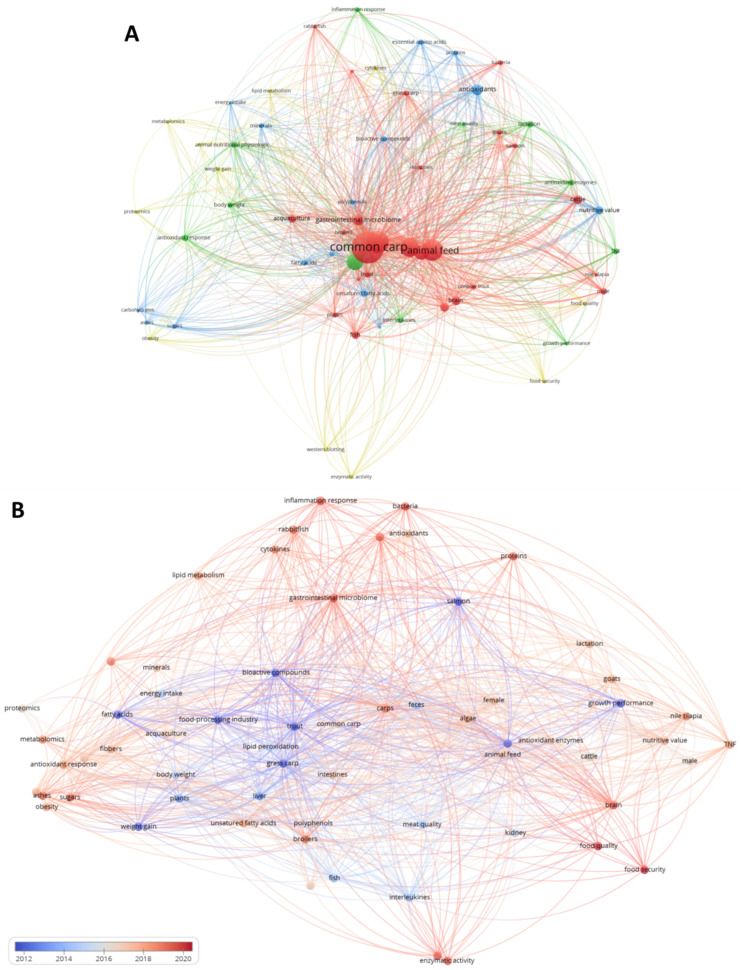
Map of the co-occurrence network of the 75 selected keywords. The prominence of the circles and texts in each cluster represents the strength of their co-occurrence, while the distance of the elements and lines shows the relation and linkage among the different keywords, respectively. (**A**) shows the grouping of keywords into four clusters, while (**B**) shows the publication period, ranging from 2012 (blue) to 2022 (red).

Bibliometric analysis is a technique aimed to understand and summarize the current state of the art of an existing or emerging research topic [30]. In this work, the data utilized for bibliometric analysis were retrieved from different scientific search engines, including Scopus, Google Scholar, ISI Web of Knowledge, and PubMed. The following keyword search string was manipulated in the search engine: ((“waste”[All Fields] OR “agri-food waste”[All Fields] OR “disposals”[All Fields] OR “by-product”[All Fields] OR “plant-based waste”[All Fields] OR “plant waste”[All Fields] AND (“diet”[MeSH Terms] OR “supplementation”[All Fields] OR “supplement”[All Fields] OR “fed”[All Fields] OR “feeding”[All Fields])) AND ((“trouts”[All Fields] OR “fishes”[All Fields] OR “carps”[All Fields] OR “salmon”[All Fields] AND “plant-based waste”[Title/Abstract]) OR “byproducts”[Title/Abstract] OR “circular economy”[Title/Abstract]) “animal feeding”[Title/Abstract] OR “aquaculture”[Title/Abstract]). Moreover, the following string (EXCLUDE “human”[All Field] OR “sheeps”[All Field] OR “pigs”[All Field] OR “chickens”[All Field] OR “rabbit”[All Field] OR “mouse”[All Field] OR “mice”[All Field]) was added in order to discard manuscripts whose aim and scope diverted from the original one. Precisely, 458 publications were retrieved by the database. Consequently, title, abstract, and keywords of each selected article were downloaded as comma-separated values (.csv) format and used for co-occurrence mapping (Figure 1A), also considering publication time as complementary factors (Figure 1B). Co-occurrence map is a semantic representation of the network subsisting among keywords of the different papers [31]. For the bibliometric study, all the terms contained in the above-mentioned sections were considered as units of analysis, but some limitations were set. Briefly, a minimum number of occurrences (*n* = 6) of a specific term was set as a limiting factor. Therefore, only 75 keywords out of 3998 articles from 458 articles met this threshold. Each keyword was analyzed by VOSviewer software (Leiden, The Netherlands), by which total link strengths and co-occurrences were calculated. Finally, the co-occurrences of the keywords were illustrated as a network visualization map. As shown in Figure 1A, the 75 keywords were able to form four different clusters: cluster 1 (red), cluster 2 (green), cluster 3 (blue) and cluster 4 (yellow). The prominence of the circles and texts in each cluster represents the strength of their co-occurrence within the other keywords, while the distance of the elements and lines shows the similarity and linkage of the keywords, respectively. The keywords in each cluster were analyzed with the aim to determine the thematic distinction of the cluster based on the distinguishable topic of the respective keywords. Cluster 1 mainly contained words related to animals used during the experiments, as evidenced by the co-occurrence of terms such as “common carp”, “grass carp”, “Nile tilapia”, and “trout”, with a greater prevalence of words referring to river or freshwater fish. This higher occurrence should not be surprising, since the control of the experimental conditions, as well as the administration of the different treatments, sampling process, or monitoring of the potential morphological changes are easier than in other experimental models.

Cluster 2 contained words mainly related to physiological and biochemical parameters measured during or after experimental treatments, such as “inflammation”, “antioxidant defenses”, and “growth performance”. Consequently, monitoring the relevant physiological processes through biochemical or genetic analysis provides important information about the health status of fish before the animal can suffer irreversible damage. Cluster 3 was composed of terms referring to the chemical composition of the plant waste used in the experiments, as evidenced by the presence of terms such as “minerals”, “fatty acids”, “bioactive compounds”, “volatile compounds”, “polyphenols”, and “proteins”. It should be specified that this cluster contains both keywords related to both nutritional and nutraceutical aspects of the plant raw material used during the experimental trials. This last finding highlights how, in recent years, there has been a growing perception that possible beneficial effects derived from food intake is not strictly and/or exclusively related to the nutritional values of food, but it may also be derived from the presence of certain biomolecules. These substances, generally called ‘phytochemicals,’ while not having a recognized nutritional status, are considered to have a biological activity of their own that can sometimes be protective or curative for the organism that consumes foods enriched with these constituents. Finally, analytical methods were mainly found in Cluster 4, along with the key terms “food quality” and “food safety”. Analyzing the same keyword network from a temporal point of view (Figure 1B), it is possible to highlight that most of the papers were published in the last five years (red connections), suggesting a growing interest in this research topic. Interestingly, the terms “food safety” and “food quality” occur as keywords most frequently after 2016, with a higher prevalence in 2021–2022. This finding suggests that scientific interest in issues related to food consumption and human health has only recently increased. This hypothesis could be further confirmed by the increased use of words such as “antioxidant response”, “inflammation”, and “lipid metabolism”.

## 4. Global Geographic Distribution and Collaboration Network of Documents Related to Use of Agri-Food Waste in Aquaculture

**Figure 2:**
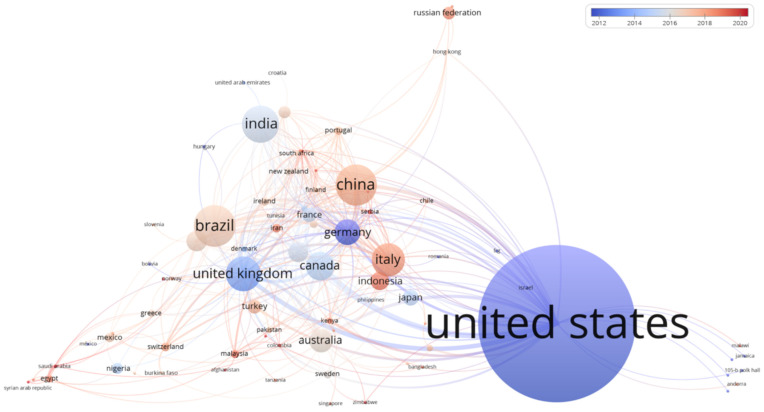
Co-authorship map showing the geographical distribution of publications over time. The prominence of the circles and texts in each cluster represents the number of publications from that country, while the distance of the elements and the connecting lines explain the relationships and potential connections between different co-authors. Finally, the different colors represent the publication period, ranging from 2012 (blue) to 2022 (red).

The 458 publications were also analyzed for a co-authorship map (Figure 2). This network visualization reports the interactions of co-authors of the previously selected articles, considering both contributing countries and time of publication. As shown in Figure 2, the United States had the largest number of published articles (*n* = 64), followed by China (*n* = 34), Brazil (*n* = 25), Italy (*n* = 22), and the United Kingdom (*n* = 20). It could be observed that high-income countries have consistently dominated the rankings in terms of scientific production, probably because they have sufficient funds and adequate tools, equipment and facilities at their disposal. In particular, the United States alone continued to dominate the scientific output impact related to this topic, reaching a total of 1248 citations and 52.1 percent of total citations, followed by China, Canada and the United Kingdom. In addition, co-authorship among countries shows that the United States has more research collaborations with researchers from China, Italy, Germany and Brazil. However, as evidenced by the different colorations of the co-authorship map (Figure 2), American publications were mostly in the years 2012–2014, while Italy, Indonesia, China, and Brazil recorded much more recent publications ranging from 2016 to 2022. In Europe, this last aspect can most certainly be attributed to the increased investment over the last few years on green and environmentally sustainable research issues. Indeed, recent events that have also affected the European community, such as a pandemic caused by COVID-19, the current economic crisis, and the war on the borders of European territory, have made clear that it is increasingly necessary for finding sustainable remedies to preserve not only the health of citizens but also that of the environment. This has achieved in an increased investment of money in public and private entities whose research was aimed at recovering waste, remediating environments, or addressing environmental pollution.

## 5. Agri-Food Waste and Growth Performance

**Figure 3:**
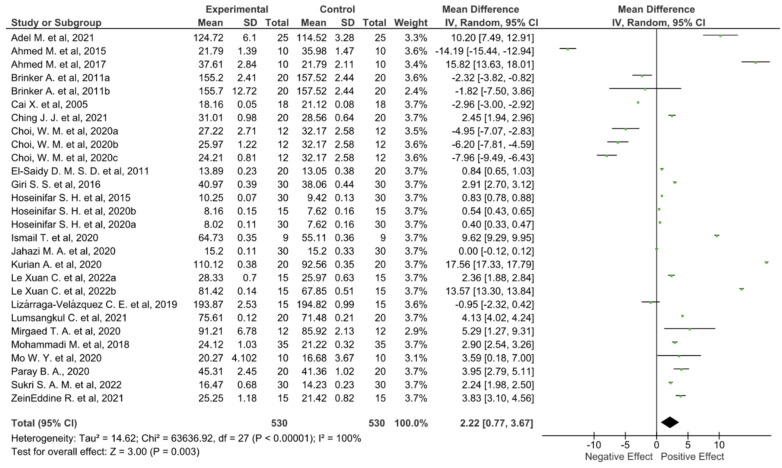
Forest diagram representation of the effects on fish growth performance resulting from supplementing fish diets with plant waste. Data were extracted from 26 articles [20,32,33,34,35,36,37,38,39,40,41,42,43,44,45,46,47,48,49,50,51,52,53,54,55,56], and plotted against the mean difference. Each horizontal line in the graph represents an individual and specific study, with the experimental mean value displayed as a green box. For each study, if the horizontal line (95% CI) crosses the vertical line (absence of effect), no statistically significant differences can be observed between the experimental and control group. The black diamond at the bottom of the forest plot represents the average effect size calculated from the combination of the results from all selected studies. The output was generated via Review Manager (RevMan) [Computer program]. Version 5.4. The Cochrane Collaboration, 2020.

Nowadays, although the price of fish food is increasingly expensive, the demand for this product is rising worldwide due to the appreciation of its nutritional value. Feeding fish by using feed that is completely or partially composed of agri-food waste may be one of the strategies to reduce the cost of fish products, also from a circular economy perspective. On the other hand, using as little feed as possible to achieve maximum fish growth performance is another important goal that should not be underestimated. To investigate whether the feeding of fish with a diet enriched by plant waste can increase aquaculture production yields, a meta-analysis on growth performance was conducted. Potential previously published articles (*n* = 232) were obtained from a literature searching using several scientific engines, namely PubMed, Scopus, Google Scholar, and ISI Web of Science. The following keywords were used for the purpose: “plant waste” OR “agri-food waste” OR “agricultural waste” OR “organic waste” AND “aquaculture” OR “fish diet” OR “fish supplementation” AND “growth performance” OR “production yield”). Then, manual screening was performed by reading the title, abstract, or full text. Original articles were included if they met the following inclusion criteria: (i) the language had to be English; (ii) the articles had to be published in peer-review journals; (iii) they had to be reviewed by scientific experts. In addition, (iv) the study design had to be a randomized controlled clinical trial involving fish commonly used in the human diet; (v) the intervention had to consist of supplementing the fish diet with plant waste; (vi) the number of fish used during the trial had to be clearly expressed, along with the measured experimental data (mean and standard deviation or standard error); (vii) the experimental parameter evaluated in the article was to be growth performance, with special emphasis on fish weight gain; (viii) when experiments involved the use of different concentrations of food waste for feed formulation, only the lowest concentration was considered to avoid toxic effects. However, when present, the other concentrations were taken into account for potential consideration during the related discussion; (ix) if the original articles reported more than one type of formulation using different plant waste, these were included as separate entries in the database. As a result, of the 232 full-text published articles, 207 were excluded. Data from the selected articles (*n* = 26) [20,32,33,34,35,36,37,38,39,40,41,42,43,44,45,46,47,48,49,50,51,52,53,54,55,56] were used for the meta-analysis (Figure 3). Because data were accumulated from a series of independently performed studies, all selected studies were not functionally equivalent. Consequently, the originated forest plots were obtained using the random effect, based on the calculated heterogeneity among the studies. Statistical heterogeneity among the studies was tested using Cochrane’s Q test (significance level *p* < 0.05) and the I^2^ statistic. In addition, a sensitivity analysis was performed with the aim to check the influence of each study on the overall effect size, while potential publication bias was verified by visual inspection of the respective funnel plots.

The combined findings of the selected articles from the random effects model suggested a slight positive effect on growth performance resulting from the feeding of fish with plant waste (WMD: 2.22; 95% CI: 0.77, 3.67; I^2^ = 100%; *p* = < 0.00001). Indeed, although five studies reported that feeding fish with plant waste could have potentially toxic effects, nineteen experimental conditions described a statistically positive effect on fish growth performance. Finally, as Figure 3 displays, the absence of a statistically significant result was never observed except for two experimental conditions [45,50]. However, the latter result can be viewed positively because the fish continued to grow normally without manifesting any toxic or side effects, while the agri-food waste was successfully removed from the environment.

Almost all studies reviewed reported toxic effects on growth performance when food waste concentrations exceeded 1–4% (*w*/*w*). This trend was observed regardless of the type of waste examined by the authors. For example, the study of Mohammadi and co-authors [51], which reported the best results on growth performance displayed in Figure 3, evaluated the effect of fish diet supplementation with palm (*Phoenix dactylifera*) seed extracts on common carp (*Eurasian carp*). The authors, during their experiment, fed carp with five diets enriched by different percentages of extract, ranging from 0.5% (*w*/*w*) to 4% (*w*/*w*) for two whole months. Contemporaneously, they carried out the same experimental conditions on fish that were normally fed. The most pronounced effects were recorded at the lowest concentration, which was shown to be able not only to increase the weight of the carp compared to control carp, but also to decrease nutrient utilization efficiency, possibly due to improved digestibility conditions of the feed in the carp’s digestive tract. Increased growth performance with the concomitant reduction in nutrient utilization efficiency is a very positive result. Indeed, as animals are more capable to assimilate nutrients, a smaller amount of nutrients must be added in feeds, consequently reducing the final cost of food products. However, the authors could not hypothesize a possible mechanism of action behind the observed morphological effects. On the contrary, negative effects on growth parameters were observed starting from the second concentration (1% *w*/*w*). The negative trend followed a dose-dependent effect, demonstrating that carp fed with 4% (*w*/*w*) palm seed extracts experienced a drastic reduction in size compared to control fish. A similar trend was observed in almost all studies that evaluated feed formulas containing different concentrations of plant waste.

Moreover, as suggested by the black diamond in the forest diagram (Figure 3), studies currently in the literature report conflicting results. This phenomenon may depend on several factors, including (i) the type of plant waste used for experiments; (ii) the concentrations of plant waste used for supplementing fish diet; (iii) the phytochemical profile of plant waste, as well as (v) its nutritional profile. Consequently, this first analysis shows that not all types of plant waste can be used for formulating fortified diets in aquaculture and that a very important factor may be the concentration of waste used for formulation. Finally, these data suggest that experimental conditions for the growth of fish fed with agri-food waste should be rigorously tested on a small scale before being used for medium to large-scale production, with the goal of finding the optimal concentration to avoid toxicological effects.

## 6. Agri-Food Waste and Immune System

**Figure 4:**
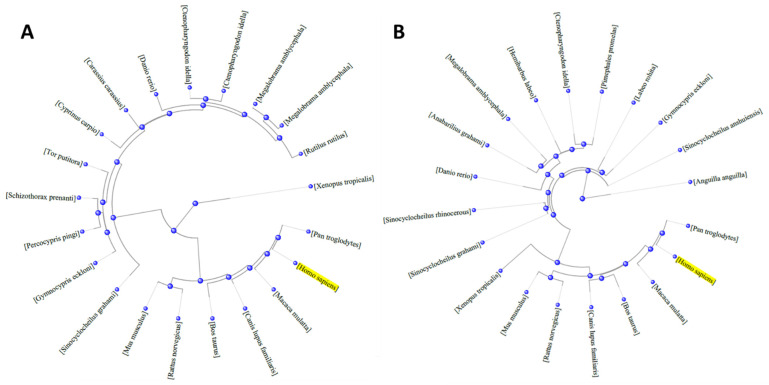
Phylogenetic tree analysis of Tumor Necrosis Factor α (TNF-α) (**A**) and Interleukin-1β (IL-1β) (**B**) originated from differences in amino acid composition of proteins identified in mammals, fish and amphibians. The circular tree demonstrates that the protein structures of mammalians is phylogenetically far respect to fish. An intermediate position is occupied by amphibians, which demonstrate sequential similarities to both mammals and fish. The protein amino acid sequences of phylogenetically close organisms are highly conserved as suggested by the link distance. The circular tree was originated by downloading the amino acid sequences from NCBI, while the distance of each entry was calculated using BLAST tool.

Inflammatory insults cause the activation of a cytokine cascade, the balance of which provokes the subsequent release of Tumor Necrosis Factor α (TNF-α). TNF-α is a member of the TNF superfamily, which consists of a series of transmembrane proteins with a homologous TNF domain. It is considered a key cytokine that regulates the complex network of pro- and anti-inflammatory cytokines. Indeed, TNF-α can trigger its own production and that of other pro- and anti-inflammatory cytokines, including IL-1β, IL-6, and IL-8. This indicates its active role not only in inflammatory processes but also in the proliferation, apoptosis, and differentiation of different cell types, such as macrophages, fibroblasts, and endothelial cells. [57]. Once released, the production of other compounds acting as chemoattractant (chemokines) is also positively modulated, whose function is inducing the migration of neutrophils and macrophages to the site of infection, thus to stimulate the inflammatory process [57,58]. TNF signaling occurs through TNF receptors (TNFRs), which are transmembrane proteins with jelly-roll structure and spatially arranged in β-sandwich conformation. This family includes some lymphotoxins, the CD40 ligand, the CD30 ligand, and the CD29 ligand [59]. While this process had long been known in mammals, little was known about the immune response to inflammatory stimuli in fish. In recent years, evidence has been collected on the existence of TNF-α in fish, but, only recently, TNF-α and TNFR genes have been cloned and identified in different fish species, including rainbow trout, common carp, catfish, zebrafish, and grass carp. Although the homology with typical mammalian TNF-α is very low (<30%), the multiplicity of TNF-α gene isoforms isolated and cloned in different fish species is very high (>85%). [60,61] (Figure 4A). The fish TNF-α cDNA consists of 1217 bp containing (i) an untranslated region of 188 bp; (ii) an open reading frame of 675 encoding the amino acid peptide; and (iii) another untranslated region of 354 bp. The untranslated regions are composed of several “TAAAT” supply motifs that, when translated into mRNA, are characterized by high instability. This characteristic is common to almost all inflammatory genes [62]. Regarding the cDNA of fish TNFR, it is 2729 bps and encodes for a protein composed of 395 amino acids. In the structure of the protein, there is a so-called “death domain”, which is also characteristic of Fas proteins. Activation of this domain follows in the initiation of all those biochemical processes that lead to apoptosis [62].

Phylogenetic analysis carried out by sequencing the genes encoding for TNF in different fish species suggests that there could be at least two different isoforms of the protein, namely TNF-α type I (TNF-α1) and TNF-α type II (TNF-α2). TNF-α2 differs substantially from the first one in the presence of a linear amino acid structure linked to the protein scaffold, whose function should be the anchoring of the TNF-α protein to the membrane. Consequently, it is believed that type I, lacking this portion, is probably the soluble version in the cellular environment. In any case, the expression of all isoforms of TNF-α is positively modulated by pro-inflammatory agents, such as LPS, peptidoglycan, polyinosinic:polycytic acid, IL-1β and IFN-γ, as well as by bacterial and viral infections [60].

With regard to IL-1β, it is a cytokine included in the β-trephile family. Genes for IL-1β have been cloned and sequenced for various fish species, which again show a large phylogenetic distance from the respective mammalian proteins (<25%). However, as shown by the circular phylogenetic tree (Figure 4B), a greater variability within fish species was also observed in comparison to TNF-α. In this case, the protein amino acid sequences share less than 45% amino acid identity [62]. Finally, analogous to TNF-α, IL-1β expression also appears to be upregulated by the exposure of the same pro-inflammatory agents [32,33,37]. In order to exert its biological function, IL-1β may bind two different receptors, IL-1R Type I (IL-1R1) and IL-1R Type II (IL-1R2). However, for the promotion of the inflammation process, the binding of IL-1β to IL-1R1 is exclusively required. In this process, similarly to mammals, the simple IL-1β/IL-1R1 protein complex is not sufficient for the activation of the inflammatory process, and the transmembrane accessory protein (IL-1RAP) must also be involved [63].

**Figure 5:**
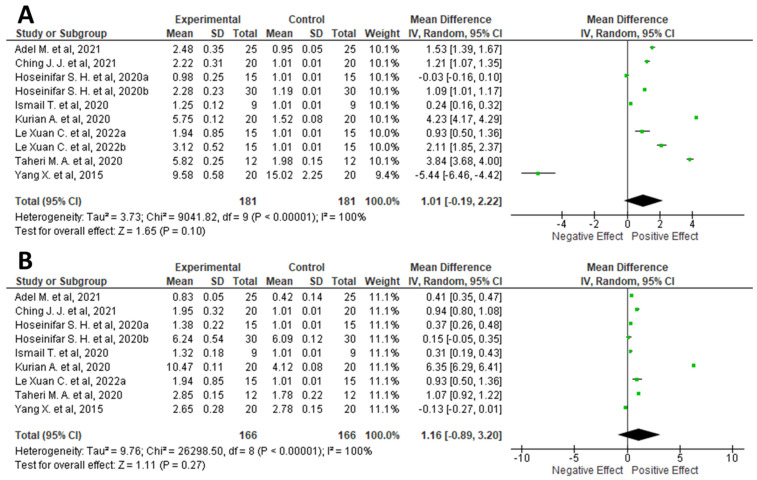
Forest diagram representation of the effects on Interleukin-1β (IL-1β) (**A**) and Tumor Necrosis Factor α (TNF-α) (**B**) resulting from supplementing fish diets with plant waste. Data were extracted from nine articles [33,34,35,36,37,38,40,42,43] and plotted against the mean difference. Each horizontal line in the graph represents an individual and specific study, with the experimental mean value displayed as a green box. For each study, if the horizontal line (95% CI) crosses the vertical line (absence of effect), no statistically significant differences can be observed between the experimental and control group. The black diamond at the bottom of the forest plot represents the average effect size calculated from the combination of the results from all selected studies. The output was generated via Review Manager (RevMan) [Computer program]. Version 5.4. The Cochrane Collaboration, 2020.

Here, in order to understand whether fish diet supplementation with plant waste could have a beneficial effect on the fish immune system, a meta-analysis was conducted. Similar to the meta-analysis conducted on fish growth performance, inclusion criteria were used to limit the search to original articles containing only data on immune responses. In addition, data collection was further limited to TNFα and IL-1β expression. Accordingly, data from selected articles (*n* = 9) [33,34,35,36,37,38,40,42,43] were used for meta-analysis (Figure 5). Because data were accumulated from a series of independently performed trials, all selected studies were not functionally equivalent. Consequently, the originated forest plots were obtained using the random effect, based on the calculated heterogeneity among the studies. Statistical heterogeneity among studies was checked using Cochrane’s Q test (significance level *p* < 0.05) and the I^2^ statistic. In addition, a sensitivity analysis was performed to test the influence of each study on the overall effect size. Finally, potential publication bias was verified by visual inspection of the respective funnel plots.

The combined results of the selected articles from the random-effects model suggested a slight positive effect on IL-1β expression (WMD: 1.01; 95% CI: −0.19, 2.22; I^2^ = 100%; *p* = < 0.00001) (Figure 5A). However, no statistical effect on TNF-α modulation (WMD: 1.16; 95% CI: −0.89, 3.20; I^2^ = 100%; *p* = < 0.00001) (Figure 5B) was observed. Regarding IL-1β, only Yang and coauthors demonstrated a highly negative effect on its release [38], whereas a statistical increase in this interleukin was reported in almost all reviewed articles. Concerning TNF-α, although Kurian and coauthors reported a highly statistical modulation of TNF-α (Figure 5B) [40], all other articles agreed on a non-statistical modulation of this protein, except for Ching, Le Xuan, and Taheri who reported just a small increment [33,34,41]. It is known that the potential increase in IL-1β and/or TNF-α correlates with an intensified innate immune system response in fish [64]. In particular, several chemically diverse compounds are successfully used in aquaculture to increase the overall resistance of fish simultaneously to a variety of infectious agents. Such compounds, known as immunostimulants, include bacterial cell wall fragments, β-1,3-glucans from yeast and mycelial fungi, peptides, and a number of synthetic products [33,65]. The main issue with the use of immunostimulants is related to both the emergence of resistance phenomena and their presence as contaminants in foods destinated human consumption [65]. The aim of immunostimulants is to increase pro-inflammatory responses of fish by the induction of cytokines. Specifically, it is fairly well established that the transcription of pro-inflammatory cytokines at a moderate level is advantageous in order to increase fish resistance against a potential infection by pathogen and to maintain immunological balance [40]. Consequently, the potential use of agri-food waste as a fish feed additive could represent a useful application in boosting fish immunity in a more natural way, avoiding the use of chemical immune stimulants, which could be harmful or potentially dangerous not only to fish but also to human health.

## 7. Agri-Food Waste and Antioxidant Defense System

Since fish are aerobic organisms, molecular oxygen (O_2_) is essential to ensure their normal and physiological functions, including metabolic processes and energy production [66]. However, equally to mammals, it has been observed that, under various metabolic conditions, aquatic organisms also fail to completely reduce O_2_ into water (H_2_O). Specifically, it was estimated that, in a mitochondrial environment, about 3–5% of O_2_ is inevitably converted into superoxide ion (O_2_^•−^) [67,68,69]. Although it has been frequently pointed out that low concentrations of O_2_^•−^ play important roles in modulating a variety of biochemical and molecular processes, the excessive production of O_2_^•−^ and other reactive oxygen species (ROS) is toxic. Indeed, in order to achieve its own stabilization, O_2_^•−^ oxidizes biological macromolecules causing extensive damage, including DNA hydroxylation, protein denaturation, lipid peroxidation, apoptosis, and eventually cell death [70,71].

However, excessive O_2_^•−^ production cannot be resolved by the simple action of soluble antioxidants. Therefore, the participation of the non-soluble fraction is essential in this process. A specific adapted enzymatic system represented by superoxidase dismutase (SOD) and catalase (CAT) has been detected in most of the fish species investigated so far [20,41,48]. SOD is the primary enzyme involved in the enzymatic detoxification of ROS. In particular, it catalyzes the reduction of O_2_^•−^ into hydrogen peroxide (H_2_O_2_). Without SOD function, this free radical leads to the formation of hydroxyl radicals (^•^OH), which are considered the most dangerous ROS because no antioxidants are available to prevent the action of ^•^OH [20,48]. However, although H_2_O_2_ is not a free radical, it can extend oxidative damage in cell cytoplasm because it can easily cross biological membranes [3,69]. Here, in the presence of transition metals such as copper and iron, H_2_O_2_ may originate further ^•^OH. In this context, CAT mediates the reduction of H_2_O_2_ in H_2_O before it becomes a potential menace for the cellular environment [69].

Living organisms, both plant and animal, have therefore learned to preserve themselves from potential oxidative insults by developing soluble as well as insoluble defense systems. For example, plants have gradually refined their secondary metabolism by producing phytochemical compounds that can not only increase their resilience to abiotic and biotic threats [3,72,73,74] but that can also act as protectants against oxidative menaces [15,16,75,76,77]. On the other hand, animals can produce soluble and endogenous compounds that, among other functions, are strong antioxidants [71,78,79]. However, unlike plants, animals are unable to produce a large amount of endogenous antioxidant molecules, both in terms of quantity and quality [78,79]. For this reason, scientific research has recently become interested in the evaluation of the antioxidant properties of plant raw materials that can be used as a potential ingredient in the supplementation of animal diet [80,81]. Indeed, as demonstrated by in vitro and clinical studies, dietary supplementation with phytochemicals can induce beneficial effects on the oxidative state of animal cells [82,83]. The main mechanisms hypothesized and described are related to (i) a direct reduction of oxidants into inoffensive species, through the radical scavenging or metal chelating properties of phytochemicals; (ii) an indirect stimulation of the transcription of genes coding for antioxidant enzymes, resulting in an increase in the amount of insoluble defenses; (iii) a direct modulation of the enzymatic activity of insoluble defenses, which are much more efficient in enzymatic detoxification even if not increased in amount [19].

**Figure 6:**
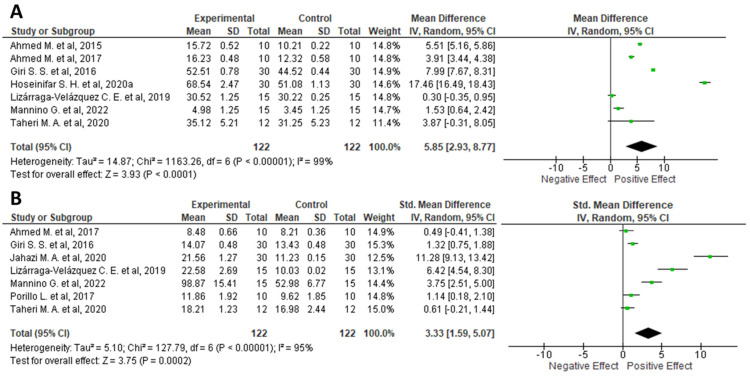
Forest plot representation of effects on Superoxide Dismutase (SOD) (**A**) and Catalase (CAT) (**B**) derived from supplementing fish diets with plant waste. Data were extracted from eight articles [20,34,44,45,46,47,48,49], and plotted according to mean difference. Each horizontal line in the graph represents an individual and specific study, with the experimental mean value displayed as a green box. For each study, if the horizontal line (95% CI) crosses the vertical line (absence of effect), no statistically significant differences can be observed between the experimental and control group. The black diamond at the bottom of the forest plot represents the average effect size calculated from the combination of the results from all selected studies. The output was generated via Review Manager (RevMan) [Computer program]. Version 5.4. The Cochrane Collaboration, 2020.

Here, in order to understand whether the supplementation of fish feed with plant waste could induce changes in the transcription of genes encoding for non-soluble antioxidant defense system or could regulate its enzyme activity, a methanalitic analysis was conducted using similar inclusion criteria to those previously described. However, the term “growth performance” was replaced with “antioxidant defense system”[All fields] AND “gene expression”[All fields] OR “enzyme activity”[All fields]. In addition, data collection was limited to SOD and CAT by adding the following search strings (“SOD”[All fields] OR “superoxide dismutase”[All fields] AND “gene expression”[All fields] OR “enzyme activity”[All fields]) or “CAT”[All fields] OR “catalase”[All fields] AND “gene expression”[All fields] OR “enzyme activity”[All fields]). Consequently, data from the selected articles (SOD: *n* = 7 [20,34,44,45,46,47]; CAT: *n* = 7 [20,34,44,45,46,48,49]) were used for the meta-analysis (Figure 6).

Because data were accumulated from a series of independently performed trials, all selected studies were not functionally equivalent. Consequently, the originated forest plots were obtained using the random effect, based on the calculated heterogeneity among the studies. Statistical heterogeneity among the studies was tested using Cochrane’s Q test (significance level *p* < 0.05) and the I^2^ statistic. In addition, a sensitivity analysis was performed to check the influence of each study on the overall effect size. Finally, potential publication biases were tested by visual inspection of the respective funnel plots. The combined results of the selected articles from the random-effect model suggested a strong and positive modulation of both SOD (WMD: 5.85; 95% CI: −2.93, 8.77; I^2^ = 99%; *p* = < 0.00001) (Figure 6A) and CAT (WMD: 3.33; 95% CI: 1.59, 5.07; I^2^ = 95%; *p* = < 0.00001) (Figure 6B). Specifically, Hoseinifar and colleagues reported the strongest modulation of SOD after supplementation of fish feed with olive waste, while Giri [44] and Jahazi [48] reported the strongest effect on modulation of CAT after supplementation of fish diet with banana and chestnut plant waste, respectively.

A clear correlation between the fish physiological state and their antioxidant defense system has been extensively described in previously scientific reports [84,85]. In particular, higher enzyme efficiency or a greater amount of non-soluble antioxidants play an effective role in mediating host benefit [85]. For this reason, synthetic antioxidants (such as butylated hydroxyl anisole and butylated hydroxyl toluene) have been widely used with the aim of increasing fish antioxidant defense levels as well as inhibiting lipid peroxidation in conventional aquaculture techniques [86]. However, the use of synthetic compounds is becoming increasingly limited because they have numerous side effects on both the environment and human or animal health [87]. Consequently, the introduction of new natural and safe substitutes that act as antioxidants is a good alternative also from a food safety perspective. The results reported in the form of forest plots in this section (Figure 6) suggest that the inclusion of agri-food waste in the diet of fish may exert a beneficial effect on the overall oxidative status of these animals, also showing greater consistency among the different experimental studies analyzed.

## 8. Conclusions

This review summarizes the potential effects of supplementing fish diets with agro-industrial waste. As it was shown, the use of agro-industrial waste for fish feed formulation is a reasonable and achievable goal, since experimental data collected to date have shown beneficial effects not only on fish growth performance but also on fish innate immune system and antioxidant defenses. However, several gaps and conflicting results suggest that not all waste can be used in the same manner in feed formulations, and that each type of by-product needs small-scale optimization before being used for an industrial scale. In addition, many of the biological effects observed during experiments on different fish species are still not fully explained through the description of the potential mechanism of action, which compromises and negatively affects the real possibility of using waste as feed additives for fish feed. However, this review shows that this goal can be achieved if future research will focus more on mechanical aspects rather than on the simple observation of morphological aspects.

## Data Availability

Not applicable.
